# Bioactive compounds, sensory attributes, and flavor perceptions involved in taste-active molecules in fruits and vegetables

**DOI:** 10.3389/fnut.2024.1427857

**Published:** 2024-09-09

**Authors:** Miriam Fabiola Fabela-Morón

**Affiliations:** Department of Food, Faculty of Chemistry, Universidad Autónoma del Estado de México, Toluca, Mexico

**Keywords:** bioactive compounds, sensory attributes, flavor perceptions, taste, fruits, vegetables

## 1 Introduction

Fruits and vegetables, as a type of food, are characterized by the presence of health-beneficial components, such as dietary fibers, antioxidants, vitamins, minerals, and bioactive compounds, which could be obtained from fruit (22%) and vegetable (27%) sources and their waste (1).

Bioactive compounds in fruits and vegetables are related to sensory attributes and flavor perceptions linked to taste-active molecules when they are consumed as part of the human diet (2, 3). Sensory attributes in fruits and vegetables including the foodstuffs and beverages derived from their processing and waste are related to taste-active molecules, which emphasize the principal flavor perceptions perceived during the handling, processing, and consumption of fruits and vegetables (4–6).

In addition, the perceived flavor in fruits and vegetables is formed by the complex interaction between taste and odor and volatile and non-volatile compounds, which act as taste-active molecules in fruits and vegetables and are responsible for flavor and sensory attributes identified in the gustatory system (7–9).

The pathways that identify the taste-active molecules signals are related to the G protein-coupled receptors, Gαi2, PLC-β2, IP_3_R3, PLA2IIa, TRPM5, KCNQ1, gustative neurons in taste bud cells, intracellular signaling, and the central nervous system (9, 10).

This article aims to present an analysis of principal bioactive compounds identified in several fruits and vegetables to recognize sensory attributes and flavor perceptions involved in taste-active molecules present in fruits and vegetables.

## 2 Bioactive compounds in fruits and vegetables

Principal bioactive compounds present in fruits and vegetables are related to taste-active molecules, being responsible for odor, flavor, aroma during growth, maturation, ripening, transformation process before consumption, and heating process, throughout anabolic and catabolic pathways, as well as autoxidation and enzymatic reactions (7). The principal bioactive compounds related to taste-active molecules in fruits and vegetables are presented in Table 1.

**Table 1:** Bioactive compounds related to taste-active molecules in fruits and vegetables.

**Bioactive compounds**	**Taste-active molecules of flavor**	**References**
Flavonoids	Rutin, chrysin, apigenin, and luteolin	(3, 8, 11)
Flavonols	Quercetin, kaempferol, myricetin, and fisetin	(3, 8, 11)
Flavanols	Proanthocyanidins, catechin, epicatechin, and epigallocatechin	(3, 8, 11)
Flavanones	Flavanone, hesperidin, naringin, and naringenin	(3, 8, 11)
Isoflavonoids	Genistein and daidzein	(3, 8, 11)
Anthocyanidins	Apigenin, malvidin, cyaniding, delphinidin, and tannins	(3, 8, 11)
Terpenes	Linalool, α-terpineol, terpineol-4-ol, steviosides, rebaudiosides, and cucurbitacins	(8, 12, 13)
Phenolic compounds	Eugenol, vanillin, apigenin, flavonoids, and coumarins	(8, 14)
Carotenoids	β-cryptoxanthin, α-carotene, and β-carotene	(8, 15)
Capsaicinoids	Capsaicin, dihydrocapsaicin, and nordihydrocapsaicin	(16)
Amino acids	Histidine, arginine, methionine, valine, leucine, isoleucine, phenylalanine, and tryptophan	(13, 17, 18)
Alkaloids	Spermine	(18)
Fatty acids	α-linoleic acid and oleic acid	(19)
Antioxidants	Ascorbic acid, dehydroascorbic acid, and β-carotene	(20)

## 3 Sensory attributes and flavor perceptions related to taste-active molecules in fruits and vegetables

**Figure 1:**
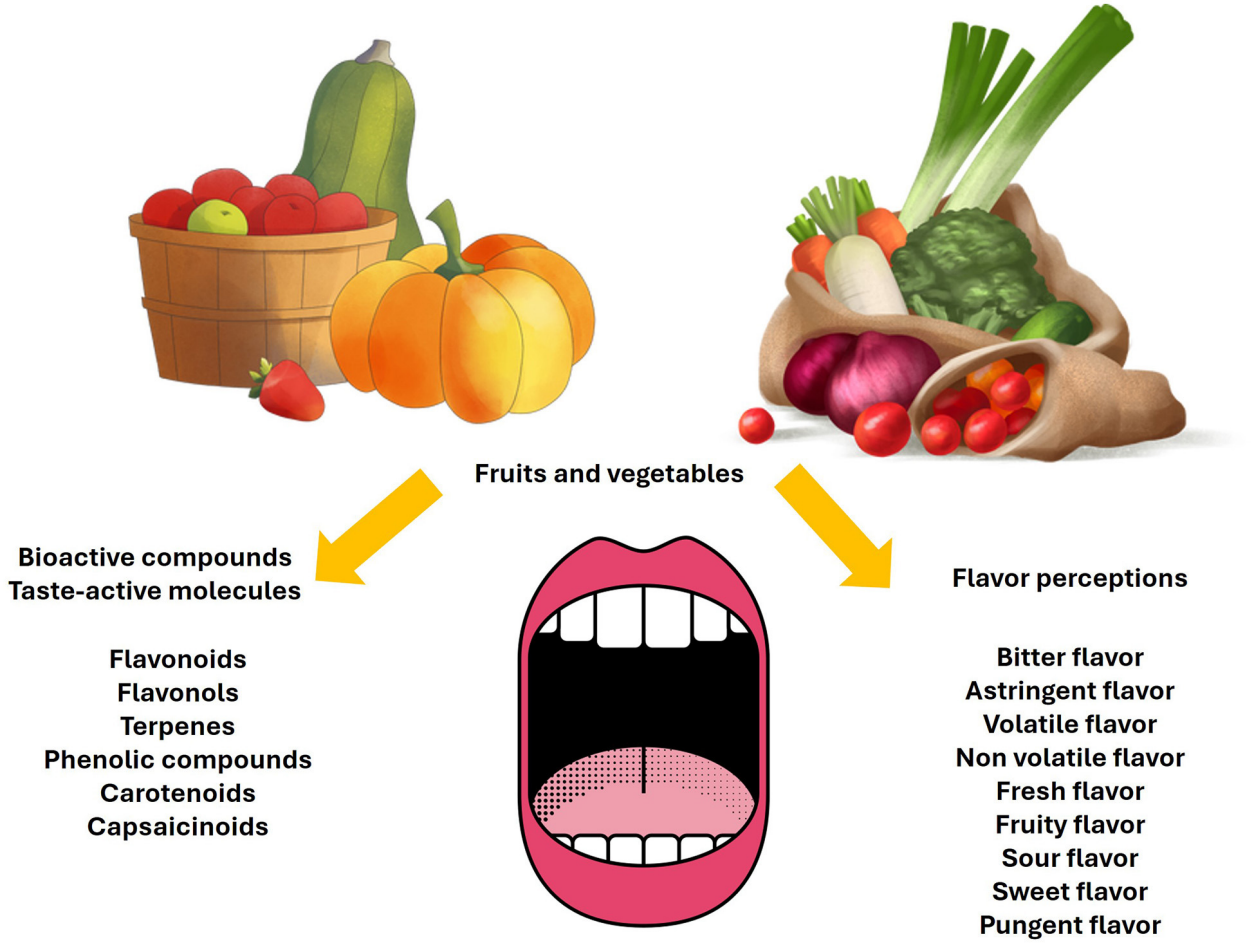
Flavor perceptions related to taste-active molecules in fruits and vegetables. Icons reproduced from icons8.com.

Flavor is a principal intrinsic property of food, being a critical motive of consumer decisions (21, 22). Bioactive compounds in fruits and vegetables provide sour, sweet, bitter, fresh, and pungent flavors, among others (8, 23, 24), which are represented in Figure 1. Therefore, sensory attributes and flavor perceptions determine the overall flavor quality in fruits and vegetables (20, 25) and influence the sensory experience of taste (10, 26, 27).

Flavor is determined by taste-active molecules throughout specialized taste receptor cells in the tongue that perceive different kinds of taste, besides the interaction of volatile constituents with the olfactory and gustative receptors (9, 19, 28). Tastings are sensed and transduced to gustatory neurons, which are finally identified in the brain as basic tastes such as sweetness, sourness, saltiness, and bitterness flavors (9, 10).

The pathways that identify the taste-active molecules signals are related to the G protein-coupled receptors, Gαi2, PLC-β2, IP_3_R3, PLA2IIa, TRPM5, KCNQ1, gustative neurons in taste bud cells, intracellular signaling, and the central nervous system. GPCR receptors are implicated in biological processes, such as neurotransmission, chemoattraction, operation of sense organs (taste, smell, and vision), and the regulation of appetite, blood pressure, and digestion. GPCR proteins, T1R and T2R as taste receptors, are ubicated on the surfaces of sensory cells in each taste bud being recognized (9, 10).

Therefore, the signals generated upon reception of taste activate the coupling G proteins and effectors, creating the stimulus in taste cells and transmitting the signals to gustatory neurons synapsed with the taste cells by means of the signal transduction-transmission process (10). On the other hand, T1R2–T1R3 receptors identify sweetness, T1R1-T1R3 perceive amino acid flavors, and T2R distinguish bitterness (9, 10, 19).

Bitter flavor is a basic taste considered disagreeable, related to phenolic compounds. In citrus fruits and juices, flavonones and neohesperidin flavonoids impart a bitter taste. Cucurbitacins are triterpenoids that provide a bitter flavor. Amino acids with hydrophobic side chains produce a bitter taste related to histidine, arginine, methionine, valine, leucine, isoleucine, phenylalanine, and tryptophan compounds. Alkaloids, such as spermine, are the substances responsible for the bitter taste in the berry fruit goji berry (13, 17, 18, 28, 29).

Astringent flavor sensation is identified as the complex of sensations between the oral epithelial cells and tannins, which arises when the oral cavities are exposed to the sensation-instigating molecules that cause a minimum of three distinct sensations inside the mouth: picked in the cheeks and face muscles, dryness in the mouth, and roughness in the oral tissues by means of physiological and psychological mechanisms on non-gustatory mucosal surfaces, including mouth friction and salivary flux (4, 30–32).

Volatile flavors involve amino acids, glucosinolates, terpenes, and phenols, which provide flowery, sweet, light, fruity, and fatty flavors in fruit varieties (19). In vegetables, volatile flavors are related to aldehydes, alcohols, ketones, esters, terpenes, and sulfur-containing compounds during the fermentation process (8, 33, 34).

Non-volatile flavors include soluble sugars (sucrose, fructose, and glucose) and organic acids (malic acid and citric acid), which are considered important indices for evaluating the flavors of fruits. These compounds are responsible for the sour, sweet, and delicious tastes of fruits (7, 8). Aldehydes and alcohols are responsible for flavors in fruits, vegetables, and green leaves related to saturated and unsaturated fatty acids, which provide fresh, green, and fruity aromas of fruits. Esters, alcohols, acids, and fatty acid carboxyl groups are produced by the oxidative degradation of linoleic acid and linolenic acid in fruits through the α, β-oxidation pathway, oxidation via the lipoxygenase pathway, and the self-oxidation pathway (8, 33). Low-carbon alcohols, aldehydes, acids, and esters in fruits and vegetables are mostly produced from amino acids through dehydrogenase, deaminase, decarboxylase, and ester synthase pathways to produce 2-methyl-1-butanol and 3-methyl-1-butanol formed during amino acid catabolism (8, 12).

Fresh flavor is associated with the ripening of fruits and vegetables, which gives a fruity flavor (sweet and intense aromatic flavors) when consumed in fresh-cut form or minimally processed, being preferred by consumers for their appealing taste, nutritional value, and healthy perception (35–37).

The sour flavor is related to organic acids (malic and citric acids), which also determine the pH levels and ripening stages of fruits and vegetables and the flavor properties of their processed products (8, 38–41).

Sweetness is considered the main factor of fruit consumer preferences attributed to the soluble sugars (glucose, fructose, and sucrose), which are key to determining their quality. Sucrose, produced by means of the glucose metabolism pathway, is converted into glucose by hexokinase and fructokinase enzymes, which are involved in the phosphorylation of fructose and glucose to produce fructose-6-phosphate and glucose-6-phosphate as characteristic sweet flavor compounds (8, 25).

The pungent flavor is associated with the degree of spiciness or heat experienced when the pepper is eaten as a natural or processed product. Pungency is provided by capsaicinoids, such as capsaicin, dihydrocapsaicin, and nordihydrocapsaicin, which are produced through phenylpropanoid and branched-chain fatty acid pathways. These compounds are responsible for the principal pungent flavor in peppers, which are used as appetite stimulants and flavoring agents. Peppers are classified as pungent (hot peppers) and non-pungent (sweet peppers) according to Scoville heat units (SHU), considering capsicum species, variety, genotype, and environmental growth conditions (16, 42–45).

## 4 Conclusion

Sensory attributes and flavor perceptions in fruits and vegetables are related to a variety of taste-active molecules that are linked to bioactive compounds such as sugars, acids, alkaloids, tannins, aldehydes, esters, ketones, alcohols, trienes, and sulfur-containing compounds. These compounds contribute to the sour, sweet, bitter, and pungent flavors perceived in the gustatory system and play a key factor in influencing consumer's sensory food choices.
